# Supplementary material for: Estimating international trade status of countries from global liner shipping networks
Source: R Soc Open Sci. 2020 Oct 14;7(10):200386. doi: 10.1098/rsos.200386 (PMC7657917; doi:10.1098/rsos.200386)
Supplement: Supplementary Information from Estimating international trade status of countries from global liner shipping networks [file rsos200386supp1.docx]

Supporting Information for

“Estimating international trade status of countries from global liner shipping networks”

Mengqiao Xu, Qian Pan, Haoxiang Xia, Naoki Masuda*

**Supplementary Table S1. Results for multivariate linear regressions when the export value, import value, net export value, or the GDP is the dependent variable and 157 countries are considered.** Gc: GLSN connectivity, Gb: GLSN betweenness, Fb: Freeman betweenness, L: LSCI. ^**^: p-value < 0.001, ^*^: p-value < 0.01, ^+^: p-value < 0.05.

| Explanatory variable | Export value | | Import value | | Net export | | GDP | | Max VIF |
| --- | --- | --- | --- | --- | --- | --- | --- | --- | --- |
|  | Adjusted $R^{2}$ | AIC | Adjusted $R^{2}$ | AIC | Adjusted $R^{2}$ | AIC | Adjusted $R^{2}$ | AIC |  |
| Gc | 0.752^**^ | -216.97 | 0.742^**^ | -210.91 | -0.006 | 2.99 | 0.674^**^ | -174.02 | 1.00 |
| Gb | 0.695^**^ | -184.42 | 0.529^**^ | -116.20 | 0.085^**^ | -11.91 | 0.306^**^ | -55.44 | 1.00 |
| Fb | 0.803^**^ | -253.26 | 0.732^**^ | -204.56 | 0.004 | 1.38 | 0.546^**^ | -121.92 | 1.00 |
| L | 0.567^**^ | -129.47 | 0.519^**^ | -112.77 | 0.000 | 1.97 | 0.288^**^ | -51.35 | 1.00 |
| Gc, Gb | 0.833^**^ | -277.74 | 0.759^**^ | -220.32 | 0.196^**^ | -31.23 | 0.678^**^ | -174.97 | 2.22 |
| Gc, Fb | 0.839^**^ | -284.17 | 0.793^**^ | -244.12 | 0.030^+^ | -1.74 | 0.677^**^ | -174.35 | 3.78 |
| Gc, L | 0.760^**^ | -220.95 | 0.743^**^ | -210.36 | 0.008 | 1.78 | 0.713^**^ | -193.12 | 2.83 |
| Gb, Fb | 0.804^**^ | -252.94 | 0.796^**^ | -246.27 | 0.411^**^ | -80.24 | 0.747^**^ | -212.79 | 9.71 |
| Gb, L | 0.712^**^ | -192.49 | 0.578^**^ | -132.36 | 0.155^**^ | -23.55 | 0.326^**^ | -58.93 | 2.87 |
| Fb, L | 0.804^**^ | -252.60 | 0.732^**^ | -203.57 | -0.003 | 3.38 | 0.555^**^ | -124.11 | 2.98 |
| Gc, Gb, Fb | 0.840^**^ | -283.83 | 0.819^**^ | -264.08 | 0.419^**^ | -81.35 | 0.782^**^ | -235.04 | 20.27 |
| Gc, Gb, L | 0.836^**^ | -279.77 | 0.758^**^ | -218.78 | 0.204^**^ | -31.90 | 0.713^**^ | -191.78 | 3.93 |
| Gc, Fb, L | 0.839^**^ | -282.98 | 0.794^**^ | -243.98 | 0.027 | -0.40 | 0.740^**^ | -207.40 | 4.55 |
| Gb, Fb, L | 0.805^**^ | -253.00 | 0.803^**^ | -250.87 | 0.424^**^ | -82.59 | 0.746^**^ | -211.17 | 10.46 |
| Gc, Gb, Fb, L | 0.841^**^ | -284.27 | 0.818^**^ | -262.37 | 0.458^**^ | -91.25 | 0.799^**^ | -246.75 | 20.95 |

**Supplementary Table S2. Coefficients for representative multivariate linear regression models, when the trade value, export value, import value, net export value, or the GDP is the dependent variable.** We considered 157 countries for year 2015. For 2017, we considered the 155 countries for which the GLSN, trade value, and LSCI were simultaneously available in the Alphaliner, World Bank [1], and UNCTAD [2] database, respectively. To readily compare the explanatory power of different regressors, we normalize the original values of the regressors and the regressend to Z-scores. The 95% confidence interval is shown in the square brackets. ^**^: p-value < 0.001, ^*^: p-value < 0.01, ^+^: p-value < 0.05.

| Dependent  variable | Explanatory variable | Coefficient | | | |
| --- | --- | --- | --- | --- | --- |
|  |  | Gc | Gb | Fb | L |
| Trade value (2015) | Gc, Fb | 0.435^**^  [0.311, 0.559] | — | 0.515^**^  [0.391, 0.639] | — |
|  | Gc, Gb | 0.643^**^  [0.541, 0.745] | 0.316^**^  [0.214, 0.418] | — | — |
| Export value (2015) | Gc, Fb | 0.374^**^  [0.251, 0.497] | — | 0.576^**^  [0.453, 0.700] | — |
|  | Gc, Gb, L | 0.612^**^  [0.500, 0.724] | 0.485^**^  [0.372, 0.597] | — | -0.128^+^  [-0.255, -0.001] |
| Import value (2015) | Gc, Fb | 0.484^**^  [0.344, 0.624] | — | 0.441^**^  [0.301, 0.581] | — |
|  | Gc, Gb | 0.715^**^  [0.599, 0.830] | 0.200^**^  [0.084, 0.315] | — | — |
| Net export (2015) | Gc, Gb, L | -0.402^*^  [-0.648, -0.156] | 0.784^**^  [0.536, 1.032] | — | -0.229  [-0.509, 0.051] |
| GDP (2015) | Gc, L | 1.095^**^  [0.953, 1.237] | — | — | -0.339^**^  [-0.482, -0.197] |
|  | Gc, Fb, L | 0.891^**^  [0.724, 1.059] | — | 0.356^**^  [0.184, 0.529] | -0.466^**^  [-0.615, -0.317] |
| Trade value (2017) | Gc, Fb | 0.419^**^  [0.284, 0.553] | — | 0.521^**^  [0.387, 0.656] | — |
|  | Gc, Gb | 0.653^**^  [0.545, 0.761] | 0.293^**^  [0.185, 0.401] | — | — |

**Supplementary Table S3. Results for multivariate linear regressions when the trade value is the dependent variable and 144 countries are considered.** ^**^: p-value < 0.001, ^*^: p-value < 0.01, ^+^: p-value < 0.05.

| Explanatory variable | Adjusted $R^{2}$ | AIC | Max VIF |
| --- | --- | --- | --- |
| Gc | 0.767^**^ | -207.53 | 1.00 |
| Gb | 0.624^**^ | -138.81 | 1.00 |
| Fb | 0.789^**^ | -222.08 | 1.00 |
| L | 0.558^**^ | -115.65 | 1.00 |
| Gc, Gb | 0.811^**^ | -236.95 | 2.21 |
| Gc, Fb | 0.838^**^ | -258.73 | 3.77 |
| Gc, L | 0.771^**^ | -209.27 | 2.81 |
| Gb, Fb | 0.813^**^ | -238.38 | 9.73 |
| Gb, L | 0.654^**^ | -150.06 | 2.95 |
| Fb, L | 0.789^**^ | -221.10 | 3.02 |
| Gc, Gb, Fb | 0.841^**^ | -260.86 | 20.63 |
| Gc, Gb, L | 0.812^**^ | -236.70 | 4.01 |
| Gc, Fb, L | 0.838^**^ | -258.16 | 4.59 |
| Gb, Fb, L | 0.817^**^ | -240.65 | 10.40 |
| Gc, Gb, Fb, L | 0.840^**^ | -259.00 | 21.51 |

**Supplementary Table S4. Results for multivariate linear regressions when the dependent variable is the trade value in 2017 and the GLSN data as well as the LSCI data in 2017 are used.** We considered the 155 countries used in Table S2. ^**^: p-value < 0.001, ^*^: p-value < 0.01, ^+^: p-value < 0.05.

| Explanatory variable | Adjusted $R^{2}$ | AIC | Max VIF |
| --- | --- | --- | --- |
| Gc | 0.757^**^ | -217.32 | 1.00 |
| Gb | 0.604^**^ | -141.45 | 1.00 |
| Fb | 0.781^**^ | -233.49 | 1.00 |
| L | 0.538^**^ | -117.63 | 1.00 |
| Gc, Gb | 0.794^**^ | -242.22 | 2.23 |
| Gc, Fb | 0.824^**^ | -265.91 | 4.05 |
| Gc, L | 0.762^**^ | -219.24 | 2.66 |
| Gb, Fb | 0.832^**^ | -273.36 | 11.53 |
| Gb, L | 0.644^**^ | -157.04 | 2.54 |
| Fb, L | 0.781^**^ | -232.49 | 2.86 |
| Gc, Gb, Fb | 0.839^**^ | -279.58 | 31.84 |
| Gc, Gb, L | 0.793^**^ | -240.42 | 3.40 |
| Gc, Fb, L | 0.823^**^ | -264.60 | 4.80 |
| Gb, Fb, L | 0.833^**^ | -273.55 | 12.99 |
| Gc, Gb, Fb, L | 0.839^**^ | -277.70 | 32.35 |

**Supplementary Table S5. Regressions of countries’ trade value change between years 2015 and 2018 on different combinations of five explanatory variables in 2015.** Tv: Trade value, Gc: GLSN connectivity, Gb: GLSN betweenness, Fb: Freeman betweenness, L: LSCI; all values are in 2015. We considered 154 countries because, among the 157 countries analyzed with the 2015 data, the trade values of 154 countries were only available in 2018 in the World Bank database [1]. ^**^: p-value < 0.001, ^*^: p-value < 0.01, ^+^: p-value < 0.05.

| Explanatory variable | Adjusted $R^{2}$ | AIC | Max VIF |
| --- | --- | --- | --- |
| Tv | 0.911^**^ | -370.12 | 1.00 |
| Gc | 0.711^**^ | -189.05 | 1.00 |
| Gb | 0.693^**^ | -180.08 | 1.00 |
| Fb | 0.790^**^ | -238.01 | 1.00 |
| L | 0.586^**^ | -133.92 | 1.00 |
| Tv, Gc | 0.910^**^ | -368.49 | 4.33 |
| Tv, Gb | 0.927^**^ | -399.23 | 2.68 |
| Tv, Fb | 0.918^**^ | -382.83 | 4.74 |
| Tv, L | 0.917^**^ | -379.54 | 2.27 |
| Gc, Gb | 0.807^**^ | -250.02 | 2.22 |
| Gc, Fb | 0.814^**^ | -255.82 | 3.77 |
| Gc, L | 0.732^**^ | -199.66 | 2.81 |
| Gb, Fb | 0.789^**^ | -236.50 | 9.69 |
| Gb, L | 0.717^**^ | -191.42 | 2.88 |
| Fb, L | 0.793^**^ | -239.94 | 2.99 |
| Tv, Gc, Gb | 0.926^**^ | -397.29 | 5.38 |
| Tv, Gc, Fb | 0.918^**^ | -381.48 | 6.23 |
| Tv, Gc, L | 0.917^**^ | -378.70 | 5.49 |
| Tv, Gb, Fb | 0.928^**^ | -400.96 | 19.25 |
| Tv, Gb, L | 0.926^**^ | -397.65 | 3.74 |
| Tv, Fb, L | 0.919^**^ | -383.98 | 6.28 |
| Gc, Gb, Fb | 0.815^**^ | -255.69 | 20.24 |
| Gc, Gb, L | 0.806^**^ | -248.27 | 3.93 |
| Gc, Fb, L | 0.813^**^ | -254.10 | 4.56 |
| Gb, Fb, L | 0.794^**^ | -239.17 | 10.43 |
| Tv, Gc, Gb, Fb | 0.928^**^ | -399.43 | 24.01 |
| Tv, Gc, Gb, L | 0.926^**^ | -396.02 | 5.57 |
| Tv, Gc, Fb, L | 0.920^**^ | -384.37 | 6.46 |
| Tv, Gb, Fb, L | 0.928^**^ | -399.63 | 19.38 |
| Gc, Gb, Fb, L | 0.813^**^ | -253.69 | 20.90 |
| Tv, Gc, Gb, Fb, L | 0.927^**^ | -397.75 | 24.56 |

**Supplementary Table S6. Multivariate regression results for gravity models in which the bilateral trade value of country pairs is the dependent variable, and the GLSN connectivity and LSBCI are two additional explanatory variables.** We considered 1818 country pairs, for which the two countries are directly connected in the GLSN (i.e., there exists at least one connection between the ports of two countries), the two countries’ GDP values are available, and the LSBCI value between the two countries is available. ^**^: p-value < 0.001, ^*^: p-value < 0.01, ^+^: p-value < 0.05.

| Explanatory variable | Adjusted $R^{2}$ | AIC | Max VIF |
| --- | --- | --- | --- |
| ln($\mathrm{GDP}_{i}\times\mathrm{GDP}_{j}$), ln($d_{ij}$) | 0.782^**^ | 1229.19 | 1.24 |
| ln($\mathrm{GDP}_{i}\times\mathrm{GDP}_{j}$), ln($d_{ij}$), ln($\mathrm{LSBCI}_{ij}$) | 0.788^**^ | 1180.62 | 2.04 |
| ln($\mathrm{GDP}_{i}\times\mathrm{GDP}_{j}$), ln($d_{ij}$), ln($\mathrm{Gc}_{i}\times\mathrm{Gc}_{j}$) | 0.788^**^ | 1176.65 | 4.35 |
| ln($\mathrm{GDP}_{i}\times\mathrm{GDP}_{j}$), ln($d_{ij}$), ln($\mathrm{LSBCI}_{ij}$), ln($\mathrm{Gc}_{i}\times\mathrm{Gc}_{j}$) | 0.789^**^ | 1165.29 | 6.29 |


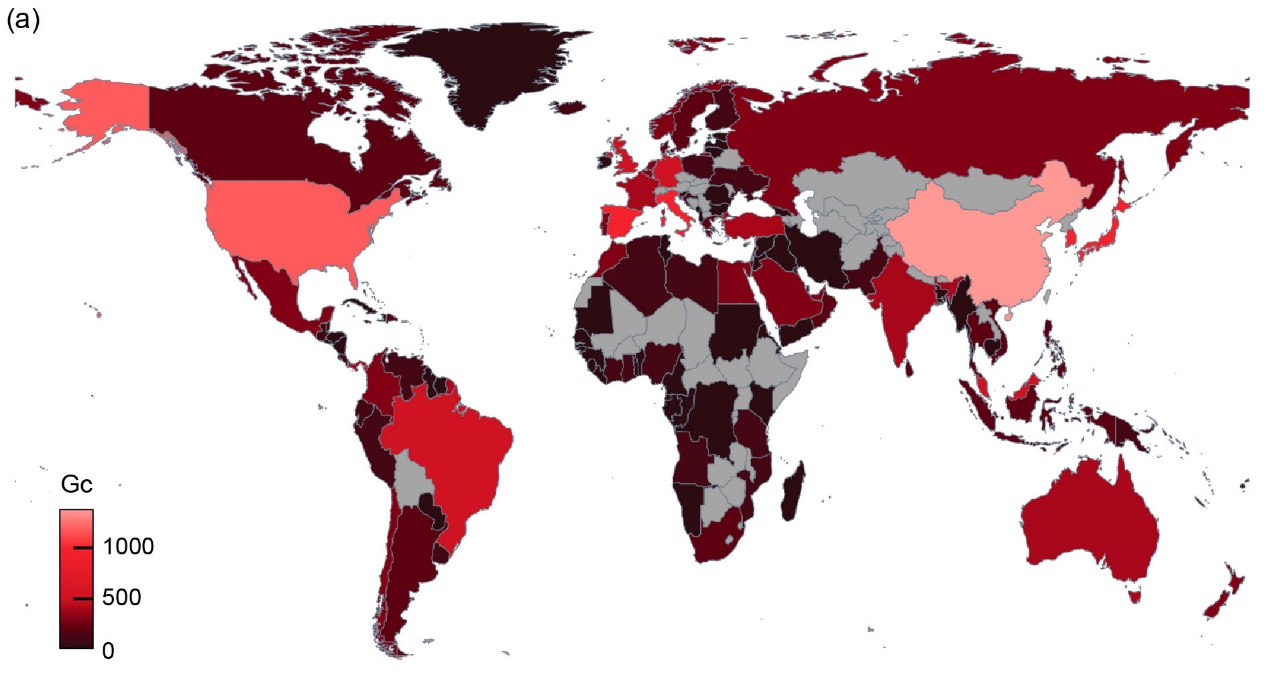


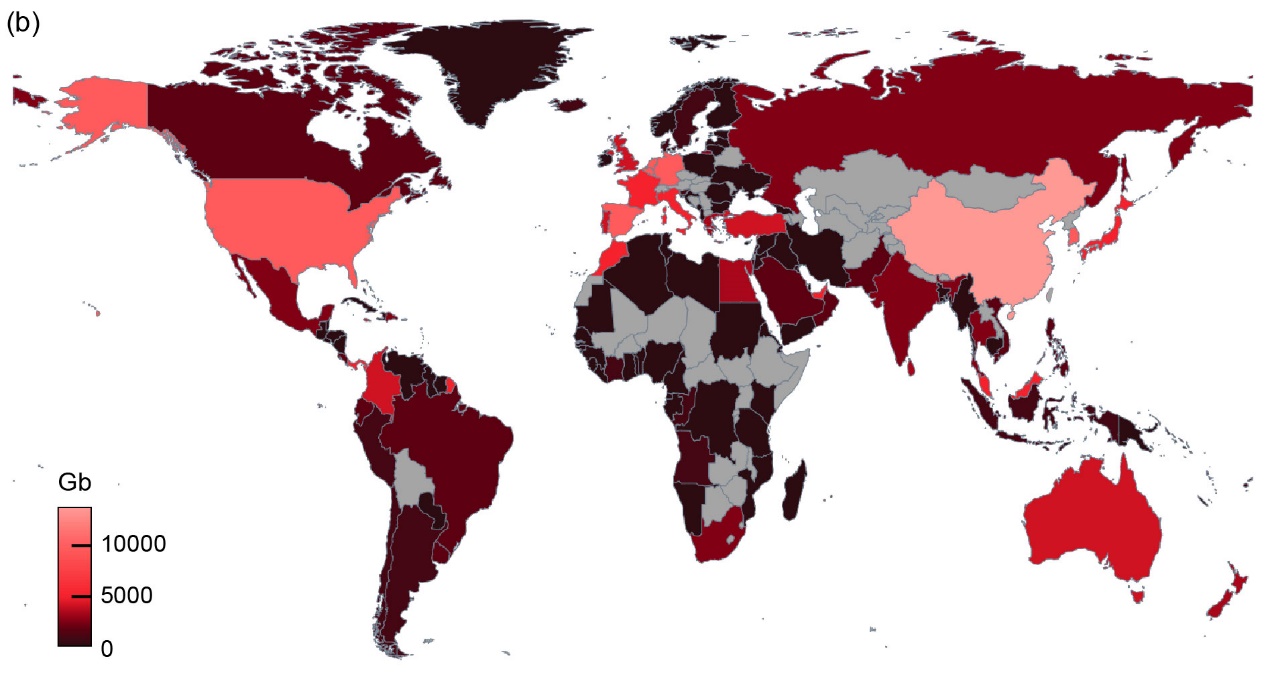


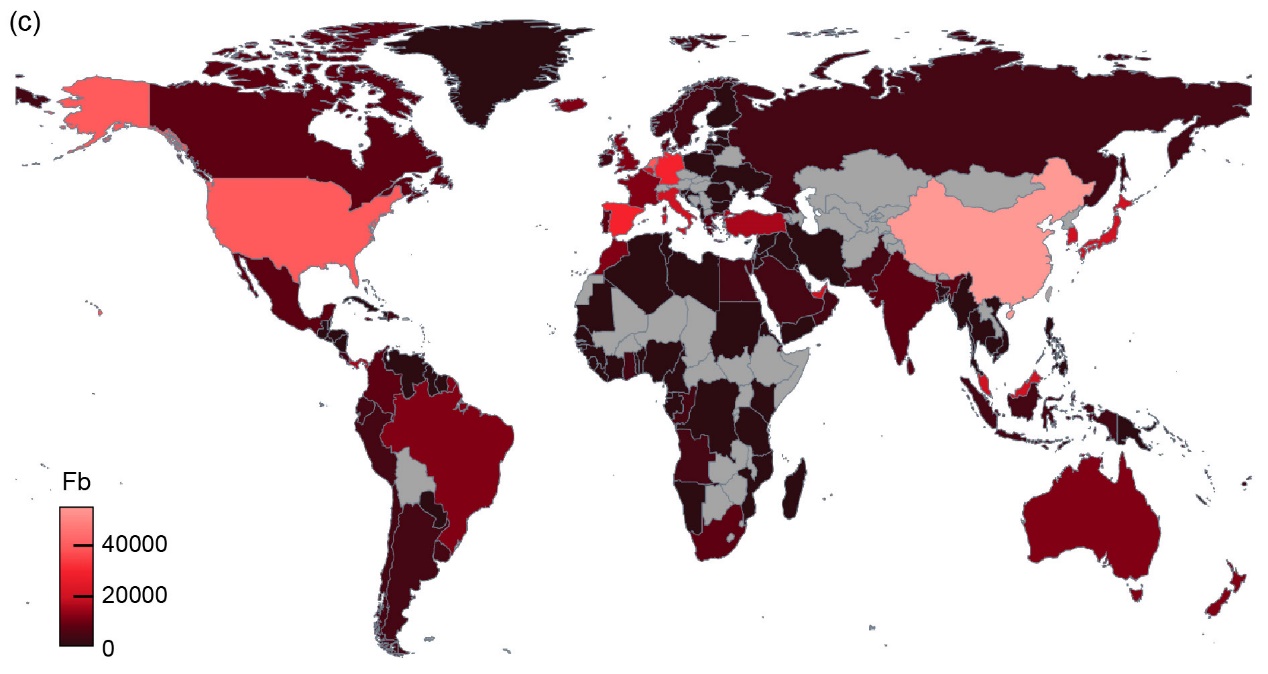


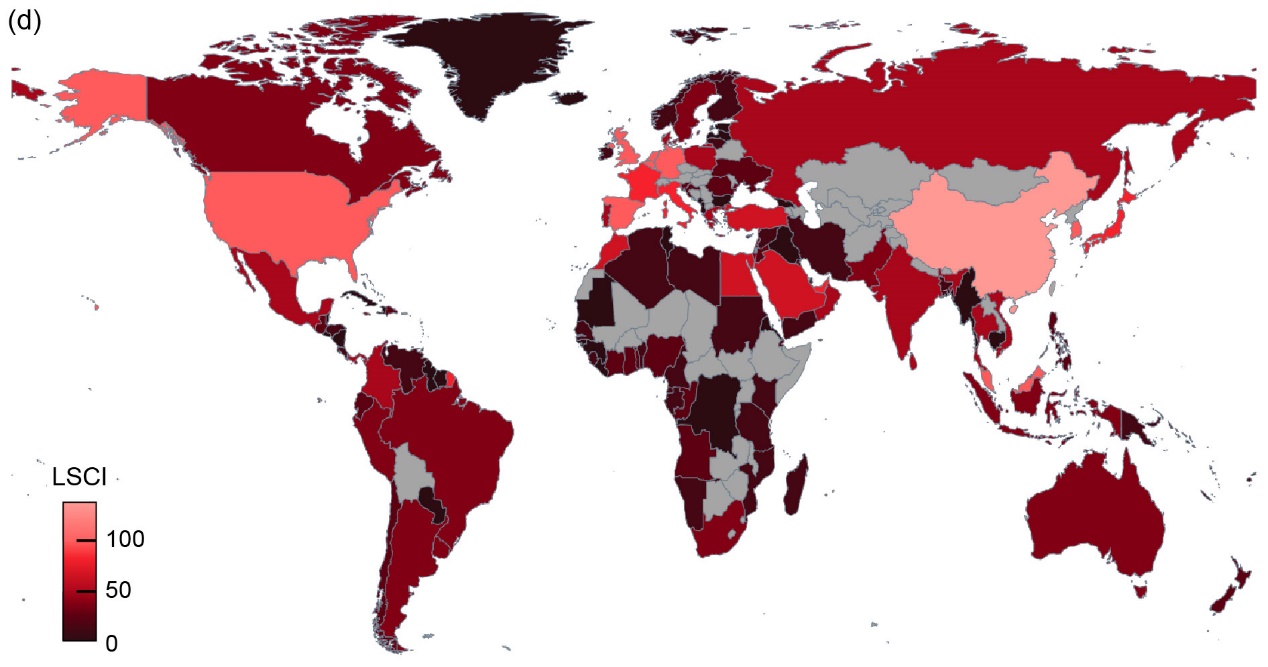


**Supplementary Figure S1. World maps showing the values of the explanatory variables for each of the 157 countries.** (a) Gc. (b) Gb. (c) Fb. (d) LSCI. The countries without the explanatory variable value are shown in grey.

**Supplementary Information References**

1. World Bank. In press. World Bank open data. See https://data.worldbank.org/ (accessed on 6 May 2019, 2 January 2020).

2. UNCTAD. In press. UNCTAD maritime transport indicators. See https://unctadstat.unctad.org/wds/ReportFolders/reportFolders.aspx (accessed on 9 August 2019, 16 July 2020).
